# Sustained Growth of the Impact Factors of MDPI Open Access Journals

**DOI:** 10.3390/molecules17021354

**Published:** 2012-02-02

**Authors:** Dietrich Rordorf

**Affiliations:** MDPI AG, Postfach, Basel CH-4005, Switzerland; Office: Kandererstrasse 25, Basel CH-4057,Switzerland; Email: rordorf@mdpi.com; Tel.: +41-61-683-77-34; Fax: +41-61-302-8918

**Figure 1:**
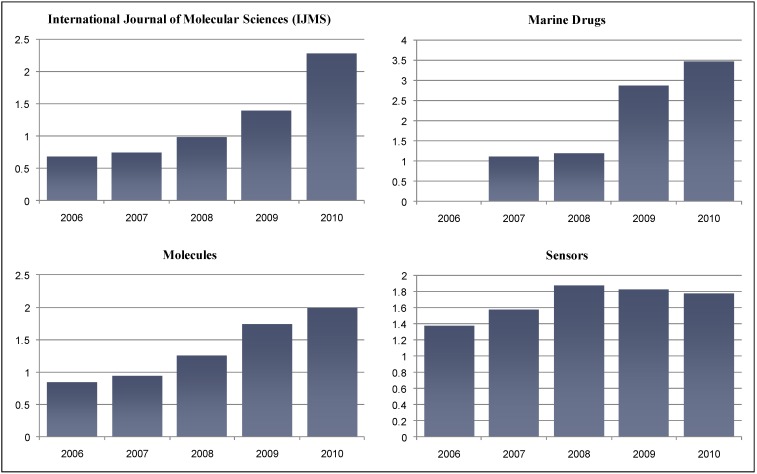
Evolution of the Impact Factors for four MDPI open access journals [adapted from the Journal Citation Reports (JCR), Edition 2010, Copyright 2011 by Thomson Reuters].

Following the tradition established during the past two years [1,2], we are pleased to report the newly released Impact Factors of MDPI open access journals by the means of an editorial. This year’s edition of the Journal Citation Reports (JCR), which is published annually by Thomson Reuters, includes seven journals published by MDPI, including three that received their first official Impact Factors – *Energies, Entropy,* and more surprisingly *Viruses* – the latest with citation data from 2009 only. We are pleased to announce that the continued growth in Impact Factors reported during the past two years has been sustained, and Impact Factors of MDPI journals continue on a growth path. Table 1 reports the latest Impact Factors for 2010. Figure 1 graphically depicts the evolution of the Impact Factors for the four MDPI open access journals that have received Impact Factors in the past. Table 2 reports the ranking of the MDPI journals within the subject categories of the Science Citation Index Expanded (SCIE).

**Table 1:** Impact Factors of seven MDPI journals [adapted from the Journal Citation Reports (JCR), Edition 2010, Copyright 2011 by Thomson Reuters].

	2006	2007	2008	2009	2010
**Energies**					1.130
**Entropy**					1.109
**IJMS**	0.679	0.750	0.978	1.387	2.279
**Marine Drugs**		1.103	1.200	2.863	3.471
**Molecules**	0.841	0.940	1.252	1.738	1.988
**Sensors**	1.373	1.573	1.870	1.821	1.771
**Viruses**					1.000

**Table 2:** Rankings of MDPI open access journals by subject categories.

Journal	Rank	Category
Energies	42/78	ENERGY & FUELS
Entropy	34/80	PHYSICS, MULTIDISCIPLINARY
IJMS	40/144	CHEMISTRY, MULTIDISCIPLINARY
Marine Drugs	9/54	CHEMISTRY, MEDICINAL
Molecules	27/56	CHEMISTRY, ORGANIC
Sensors	14/61	INSTRUMENTS & INSTRUMENTATION
	16/26	ELECTRO–CHEMISTRY
	38/71	CHEMISTRY, ANALYTICAL
Viruses	29/32	VIROLOGY

The sustained and continued growth of the Impact Factors of MDPI journals provides further evidence for the citation advantage of the full open access policy, which was instituted for all MDPI journals in early 2007 [3,4].
